# Total Hip Arthroplasty for Implant Rupture after Surgery for Atypical Subtrochanteric Femoral Fracture

**DOI:** 10.1155/2016/7146419

**Published:** 2016-10-12

**Authors:** Yu Ozaki, Tomonori Baba, Hironori Ochi, Yasuhiro Homma, Taiji Watari, Mikio Matsumoto, Kazuo Kaneko

**Affiliations:** Department of Orthopedic Surgery, Juntendo University School of Medicine, 2-1-1 Hongo, Bunkyo-ku, Tokyo, Japan

## Abstract

Treatment methods for delayed union and nonunion of atypical femoral fracture are still controversial. Moreover, no treatment method has been established for implant rupture caused by delayed union and nonunion. We encountered a 74-year-old female in whom nonunion-induced implant rupture occurred after treatment of atypical subtrochanteric femoral fracture with internal fixation using a long femoral nail. It was unlikely that sufficient fixation could be obtained by repeating osteosynthesis alone. Moreover, the patient was elderly and early weight-bearing activity was essential for early recovery of ADL. Based on these reasons, we selected one-stage surgery with total hip arthroplasty and osteosynthesis with inverted condylar locking plate as salvage procedures. Bone union was achieved at 6 months after surgery. This case illustrated that osteosynthesis-combined one-staged total hip arthroplasty could be considered as one of the options for nonunion-induced implant rupture of atypical femoral subtrochanteric fracture.

## 1. Introduction

Reportedly, delayed union and nonunion are likely to occur after treatment of atypical femoral fracture compared with those after normal fracture [1, 2]. However, treatment methods for delayed union and nonunion of atypical femoral fracture are still controversial. Moreover, no treatment method has been established for implant rupture caused by delayed union and nonunion [2, 3]. We encountered a patient in whom implant rupture occurred after treatment of atypical subtrochanteric femoral fracture with internal fixation using a long femoral nail. We treated this patient with invasive osteosynthesis-combined one-stage total hip arthroplasty.

## 2. Case Report

**Figure 1:**
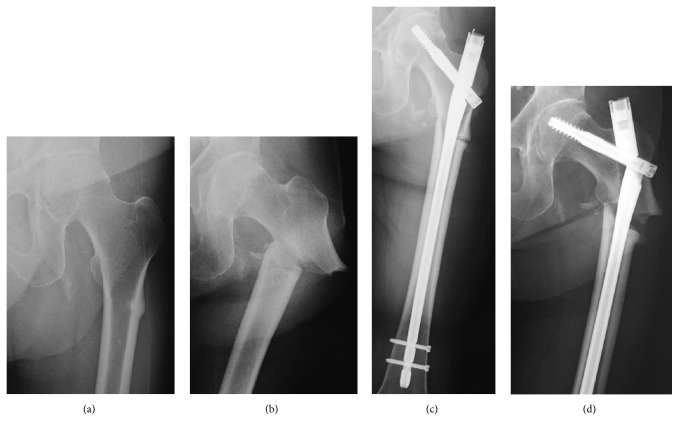
(a) An AP radiograph of the left femur shows a transverse fracture line and thickening of the lateral cortex at the subtrochanteric area. (b) A completely displaced fracture. (c) The internal fixation using a long femoral nail. (d) The nail breakage and opening of the fracture region.

**Figure 2:**
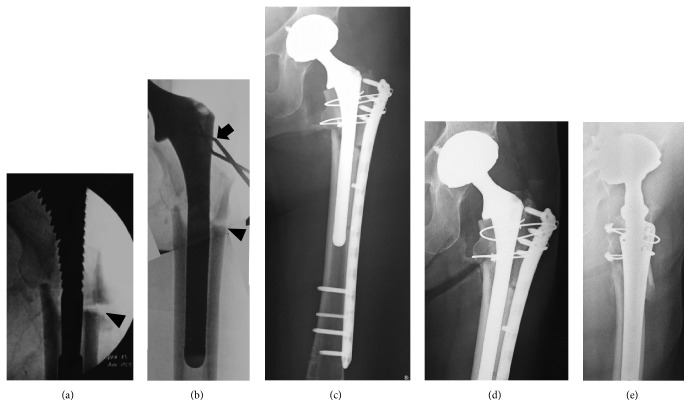
(a) A trial broach with the optimum size was inserted, but reduction was not enough (arrowhead). (b) Varus of the proximal bone fragment was prevented using the Blocking Pin Technique (arrow), and the stem was placed in the optimal position (arrowhead). (c) The osteosynthesis-combined one-staged total hip arthroplasty. (d) AP and lateral views on radiograph obtained after second surgery six months show complete union of the fracture.

A 74-year-old female had a past medical history of Adult Still's disease and she had been treated with steroids for 23 years at the Department of Collagen Disease. Treatment of osteoporosis with bisphosphonates was initiated 4 years ago and the drug was switched to denosumab 1 year ago. She felt pain in the left femoral region 3 months before injury and underwent plain radiography. Breaking and a slightly radiolucent line were observed in the lateral subtrochanteric femoral cortex, and she was diagnosed with atypical incomplete subtrochanteric femoral fracture (Figure 1(a)). Then we measured bone turnover markers. Bone resorption and bone formation markers were suppressed (TRAP5b: 154 mU/dL, BAP: 11.2 *μ*g/L, and PINP: 11.2 ng/mL), and serum levels of calcium (9.2 mg/dL) and phosphate (3.2 mg/dL) and the concentration of intact parathyroid hormone (65 pg/mL) were normal. Conservative treatment was planned, the drug was changed from denosumab to teriparatide, and she started walking with 2 crutches. There was a reduction in pain, but she fell and was unable to move her body, so she was transported to our hospital by ambulance. On plain radiography, transverse fracture accompanied by a spike was noted on the medial side. Atypical subtrochanteric femoral complete fracture was diagnosed (Figure 1(b)), and invasive osteosynthesis with a long femoral nail (Gamma3 long nail, Stryker) was performed (Figure 1(c)). Full weight-bearing exercise was applied as much as possible in treatment following surgery. Gait with a T-shape cane became stable and the patient was discharged 1 month after surgery. However, left thigh pain appeared again 2 months after surgery, and nail breakage and opening of the fracture region due to delayed union were observed on plain radiography (Figure 1(d)). In reoperation, the implant was removed, and total hip arthroplasty through the posterior approach was performed. For the acetabular component, a dual mobility cup (MDM X3, Stryker) was used. For the femoral side, a distal fixation-type long stem (Restoration HA, Stryker) was used. As a modification of the surgery, varus of the proximal bone fragment was prevented using the Blocking Pin Technique (*φ*2.4 mm Kirschner wires) (Figures 2(a) and 2(b)) [4], and the stem was placed in the optimal position. The resected autologous femoral head was trimmed and transplanted into the bone defect formed between the screw insertion region and nonunion region. To fix the nonunion region, a condylar locking plate (LCP-DF, Synthes) for the opposite side was inverted and used, and osteosynthesis was also applied (Figure 2(c)). Low-Intensity Pulsed Ultrasound (LIPUS) treatment was initiated immediately after surgery. At the same time, administration of a PTH preparation was initiated. Weight-bearing exercise was initiated one week after surgery as treatment following surgery. She became able to walk with a cane 2 months after surgery and was discharged. Callus formation was observed 5 months after surgery and bone union was achieved at 6 months. Although radiolucent line at the lateral femoral cortex can still be seen, there was the callus formation in the three directions except for the lateral cortex (Figures 2(d) and 2(e)). As of 2 years after surgery, there has been no problem with the implant (total hip arthroplasty and materials of osteosynthesis), and the patient's physical activity level returned to that before injury.

## 3. Discussion

In subtrochanteric femoral fracture, abduction, bending, and external rotation of the proximal bone fragment occur and adduction and shortening of the distal bone fragment occur due to strong muscles attaching to the region around the hip joint. Additionally, acquisition and retention of a favorable reduction position are difficult. Moreover, weight-bearing loads apply tension to the lateral bone cortex and pressure to the medial bone cortex in this region, resulting in a large load of stress concentration applied on the implant [5]. In addition to deterioration of bone quality, an influence of microdamage accumulation, due to remodeling failure, in atypical femoral fracture has been observed [6, 7]. When injury reaches complete fracture, many complications occur even if it is surgically treated, and the risk of delayed union/nonunion and accompanying implant rupture is high [1]. Atypical subtrochanteric femoral fracture is difficult to treat because it has properties of both subtrochanteric femoral fracture, which is biomechanically disadvantageous for bone union, and atypical femoral fracture, which is biologically disadvantageous.

In this patient, conservative treatment was selected when incomplete atypical subtrochanteric femoral fracture was diagnosed, but poor prognosis of conservatively treated incomplete fracture has been occasionally reported [8, 9]. Considering the difficulty of surgery for complete atypical subtrochanteric femoral fracture, preventive internal fixation is recommended when incomplete fracture is diagnosed [8–10]. We recognized that surgery, not conservative treatment, should be indicated even though the fracture is incomplete when precursor pain is observed and a radiolucent line is observed in addition to breaking [6, 7, 11, 12].

There is room for discussion about whether osteosynthesis should be reapplied or an arthroplasty should be used for salvage procedures of nonunion-induced implant rupture. To our knowledge, no report recommended one-stage surgery with an arthroplasty. In the present patient, a radiolucent line extended widely from the nail breakage region to the lag screw insertion region, and exchange of the intramedullary nail was likely to be insufficient for initial fixation. In addition, poor outcomes of fixation of atypical femoral fracture with a plate alone have occasionally been reported [1, 13]. Thus, it was unlikely that sufficient fixation could be obtained by repeating osteosynthesis alone. Moreover, the patient was elderly and early weight-bearing activity was essential for early recovery of ADL. Based on these reasons, we selected the combination of total hip arthroplasty and osteosynthesis as salvage procedures. The stem selected for the femoral side was a distal fixation-type long stem with an extensive porous coating so that a bypass is formed across the fracture region, aimed at early weight-bearing activity [14]. For the acetabular component, considering that the surgery was a reoperation in an elderly patient, the dual mobility cup was used to prevent dislocation [15]. To strongly fix the fracture region, condylar LCP was inverted and used for internal fixation because many screws can be inserted into the proximal bone fragment using this plate [16, 17]. Bone transplantation as salvage surgery for atypical fractures is reported to be effective, and we also collected cancellous bone from the excised femoral head and transplanted it to the bone defect region [2]. Promotion of fracture healing by PTH preparation alone or in combination with LIPUS in atypical femoral fracture has been reported, so we adopted it [18–20].
